# Study on the Optimization of Mix Proportions for Recycled Aggregate Concrete and Its Freeze–Thaw Resistance Performance

**DOI:** 10.3390/ma19091683

**Published:** 2026-04-22

**Authors:** Ping Zheng, Wei Deng, Wenyu Wei, Chao Pu, Zhiwei Yang, Bing Ma, Jialong Sheng, Peng Yin

**Affiliations:** 1 Xinjiang Key Laboratory for Safety and Health of Transportation Infrastructure in Alpine and High-Altitude Mountainous Areas, Urumqi 830011, China; 2 Xinjiang Transport Planning Survey and Design Institute Co., Ltd., Urumqi 830006, China; 3 Xinjiang Road and Bridge Construction Group Co., Ltd., Urumqi 830011, China; 4 School of Civil Engineering, Xinjiang Institute of Engineering, Urumqi 830023, China; 5 School of Infrastructure Engineering, Dalian University of Technology, No. 2, Linggong Road, Ganjingzi District, Dalian 116024, China; 6 Key Laboratory of Ecological Safety and Sustainable Development in Arid Lands, Xinjiang Institute of Ecology and Geography, Chinese Academy of Sciences, Urumqi 830011, China; 7 University of Chinese Academy of Sciences, Beijing 100049, China

**Keywords:** recycled aggregate concrete, orthogonal experiment, mix design optimization, freeze–thaw cycles, damage variable model

## Abstract

The growing volume of construction and demolition waste has made discarded concrete a major source of urban solid waste, placing increasing pressure on land resources and the environment. Recycling waste concrete into recycled aggregate concrete (RAC) offers an effective solution for resource conservation and carbon reduction, aligning with the goals of sustainable development. However, due to the residual mortar, high porosity, and microcracks of recycled aggregates, RAC generally exhibits lower compactness, strength, and durability than conventional concrete, particularly under freeze–thaw conditions where degradation accelerates and service life decreases. To address these challenges, this study investigates the optimization of RAC mix design and its frost resistance performance for pavement base applications. An orthogonal experimental design was employed, with the water-to-binder ratio, recycled aggregate replacement ratio, and air-entraining agent dosage as key variables, while 7-day compressive strength, permeability coefficient, and rebound modulus served as evaluation indices. The influence and interaction of these factors were analyzed to determine an optimal mix meeting both mechanical and durability requirements. Rapid freeze–thaw cycling tests were then conducted to examine the variations in mass loss, relative dynamic modulus, and compressive strength retention, followed by exponential and damage variable modeling to characterize the degradation process. Results show that the water-to-binder ratio primarily governs strength, the replacement ratio affects stiffness and permeability, and the air-entraining agent significantly enhances frost resistance by improving pore structure. The optimized mix retained over 70% of its relative dynamic modulus after 300 freeze–thaw cycles, exhibiting superior durability. This work establishes a systematic framework for multi-factor optimization and durability evaluation of RAC, providing theoretical and practical guidance for its application in cold-region pavement bases.

## 1. Introduction

With rapid urbanization and large-scale infrastructure renewal, enormous quantities of waste concrete are generated from the demolition and reconstruction of aging buildings and pavements. Construction and demolition waste has become a major component of global solid waste, and its uncontrolled accumulation not only consumes valuable land resources but also increases dust, leachate pollution, and carbon emissions, posing a serious threat to environmental sustainability [1,2]. In response to carbon reduction and circular economy goals, recycling waste concrete into recycled aggregate concrete (RAC) provides an effective means of resource recovery and green material utilization. By partially or fully replacing natural aggregates with processed recycled aggregates, RAC can significantly reduce the demand for natural sand and gravel while mitigating the environmental burden of waste disposal. However, the residual mortar, high porosity, and microcracks inherent in recycled aggregates lead to lower density, strength, and durability compared with conventional concrete [3,4]. These weaknesses become more pronounced under severe conditions such as freeze–thaw cycles, wet–dry alternation, and hydraulic erosion, resulting in accelerated deterioration and limiting the broader engineering application of RAC [5].

To address the inherent deficiencies of RAC, extensive studies have been carried out worldwide. Research has shown that a well-designed mix proportion can partially mitigate the strength and durability degradation caused by recycled aggregates [6,7]. Incorporating supplementary cementitious materials such as fly ash, slag powder, and silica fume can refine the matrix structure, enhance the interfacial transition zone (ITZ), and improve both mechanical performance and resistance to aggressive environments [8,9]. In addition, the use of air-entraining agents and high-range water reducers has been proven to reduce porosity and enhance the freeze–thaw and permeability resistance of RAC [10,11]. Meanwhile, modification treatments targeting the recycled aggregates themselves—such as pre-soaking, carbonation curing, and surface coating—have been developed to lower water absorption and strengthen the bond with the cement paste, thereby improving the overall durability of RAC [12,13]. For example, Xuan [14] reported that carbonation-treated RAC significantly reduced water absorption and permeability, resulting in enhanced durability. Limbachiya [15] demonstrated that replacing a high proportion of natural coarse aggregates with RAC in combination with fly ash maintained satisfactory mechanical and durability performance that outperforms conventional concrete and meets the strict technical requirements for pavement bases. Specifically, the optimized RAC exhibits higher compressive strength and rebound modulus than conventional concrete, along with better freeze–thaw resistance and lower water permeability—performance characteristics that are comparable to or even superior to those of natural aggregate concrete used in practical pavement engineering. Wang [16] found that the replacement of cement with a blend of silica fume, slag, and fly ash significantly affected compressive strength, and the synergy among these materials markedly enhanced overall performance. Sasanipour [17] evaluated RAC pretreated with silica-fume slurry and observed that this method substantially improved both durability and ITZ quality. Yu [18] prepared self-compacting concrete incorporating RAC and superplasticizers, achieving mechanical properties comparable to those of conventional concrete. Matar [19] further investigated the combined use of RAC and polypropylene fibers in self-compacting concrete, revealing that while fibers reduced flowability, RAC contributed to better structural stability. Despite these advances, most existing studies remain limited to single-factor analyses and lack systematic understanding of the coupled effects among key parameters governing the mechanical behavior and durability evolution of RAC. Moreover, most current research focuses primarily on compressive strength under standard conditions, with relatively few investigations into long-term performance under complex service environments such as freeze–thaw cycles. This gap has hindered the broader application of RAC in semi-rigid and rigid pavement base courses and other infrastructure exposed to severe climatic conditions.

In summary, although considerable progress has been made in mix design optimization, interfacial structure improvement, and durability enhancement of RAC, existing studies still exhibit limitations such as narrow research perspectives, incomplete performance evaluation systems, and insufficient attention to complex service environments. In particular, the coupled deterioration mechanism of RAC’s mechanical and durability properties under freeze–thaw cycles has not yet been systematically elucidated, and practical quantitative methods for design optimization remain lacking. Therefore, it is essential to establish a comprehensive multi-factor regulation and evaluation framework to reveal the performance evolution of RAC under complex environmental conditions and to achieve both mix proportion optimization and intrinsic improvement of service performance. Based on this rationale, this study focuses on RAC used in semi-rigid and rigid pavement base courses, aiming to optimize its mix design and enhance frost resistance. An orthogonal experimental design incorporating multiple factors and performance indices was employed to investigate the interactive effects among water-to-binder ratio, recycled aggregate replacement ratio, and admixture content. Furthermore, rapid freeze–thaw cycling tests were conducted to evaluate the mechanical and durability evolution of RAC. The findings are expected to provide both theoretical guidance and practical reference for the application of RAC in pavement engineering, contributing to solid waste reutilization and the development of sustainable construction materials.

## 2. Materials and Methods

### 2.1. Materials

Ordinary Portland cement (P·O 42.5) was used as the primary binder, and its chemical composition and physical properties complied with the specifications of GB 175–2020 [20]. The fine aggregate was medium river sand with a fineness modulus of 2.6 and a mud content below 2%, meeting the requirements of 3% of JTG E42–2005 [21]. Two types of coarse aggregates were employed: natural crushed stone with a particle size range of 5–26.5 mm, a flaky particle content of 8%, and a crushing value of 15%; and recycled coarse aggregate (RCA) derived from waste C30 concrete through crushing, sieving, and secondary washing. The RCA exhibited an apparent density of 2.45 g/cm^3^, water absorption of 5.8%, a flaky particle content of approximately 12%, and about 30% adhered mortar, satisfying the requirements of GB/T 25177–2010 [22]. To reduce the influence of surface defects on concrete performance, the RCA was pre-soaked to saturation and surface-dried to achieve a saturated surface dry condition before mixing, minimizing water absorption during batching. A lignosulfonate-based air-entraining agent with a solid content of 5% was used to enhance frost resistance, while Class II fly ash with a specific surface area of 420 m^2^/kg and a water demand ratio of 95%, conforming to GB/T 1596–2017 [23], served as the supplementary cementitious material. All raw materials met the technical specifications outlined in JTG/T F20–2015 [24], and their properties is shown in Table 1.

### 2.2. Specimen Preparation Process

Based on the relevant studies [25], the concrete mix proportions were designed based on the volumetric method, with three main control variables: the water-to-binder ratio (0.35, 0.38, and 0.42), the replacement ratio of recycled coarse aggregate (30%, 50%, and 70%), and the dosage of air-entraining agent (0.02%, 0.04%, and 0.06% by mass of binder). The specimen preparation procedures followed the guidelines of JTG E30–2005 [26]. Prior to mixing, the recycled coarse aggregates were washed and pre-soaked in water for 24 h, then air-dried to a saturated surface dry (SSD) condition to minimize water absorption during mixing. Mixing was conducted using a planetary laboratory mixer (YXQM-2L) from Guangzhou Gurui Technology Co., Ltd., Guangzhou, China. Coarse and fine aggregates were first dry-mixed for 30 s to ensure uniform distribution, followed by the addition of cement and fly ash for an additional 60 s of dry mixing. The air-entraining agent solution and mixing water were then gradually introduced, and mixing continued for another 120 s until a uniform mixture was achieved. After mixing, a slump test was immediately conducted, and the workability was controlled within 60–80 mm to ensure proper compaction and molding.

The specimens were cast using a combination of internal and external vibration to achieve adequate compaction and air release. Cylindrical steel molds with a diameter of 150 mm and a height of 150 mm were used. After casting, the specimens were covered with plastic film to prevent early moisture loss and were demolded after 24 h of ambient curing. The demolded specimens were then placed in a standard curing chamber at a temperature of (20 ± 2) °C and relative humidity above 95% until the designated testing age.

### 2.3. Methods

#### 2.3.1. Mix Proportion Optimization Test

To systematically investigate the influence of various factors on the performance of RAC, the 7d compressive strength (Rc), permeability coefficient (K), and rebound modulus (Ec) were selected as comprehensive evaluation indices. The selection of Rc, K, and Ec as core evaluation indices for RAC mix performance is grounded in the functional demands of pavement base courses, intrinsic properties of RAC, and compliance with engineering standards. Rc is critical for guiding pavement base construction scheduling (enabling timely formwork removal and subsequent operations) and aligns with JTG/T F20–2015’s [24] on-site quality control requirements, while also pre-judging long-term mechanical stability of RAC (which is prone to early performance fluctuations due to RCA’s high water absorption). K targets durability—a key concern for cold-region pavements—by characterizing matrix density: lower K reduces free water ingress, mitigating freeze–thaw damage (the primary durability failure mode in low temperatures) and meeting GB/T 50082–2009’s [27] durability criteria. Ec reflects stiffness and deformation resistance, which are mandatory for pavement structural design (to avoid cracking/rutting under repeated traffic loads) and addresses RAC’s inherent stiffness deficiency caused by RCA’s porosity. Together, these indices comprehensively cover construction feasibility, durability, and structural performance, forming a targeted evaluation framework for optimizing RAC mixes for cold-region pavement bases.

Moreover, the permeability coefficient was determined in accordance with GB/T 50082–2009 [27]. The specific procedure is as follows: first, cure the 150 mm × 150 mm cylindrical specimens to 28 days of age; then seal the side surfaces of the specimens with waterproof coating to ensure water penetration only through the upper and lower surfaces; place the sealed specimens in a permeability tester, apply a constant water pressure of 0.1 MPa, and maintain the pressure for 24 h to stabilize the seepage state; record the cumulative water seepage volume within 60 min after stabilization; finally, calculate the permeability coefficient using the formula for steady-state water seepage in porous materials, considering the specimen dimensions, water pressure and seepage time. The rebound modulus test was conducted in accordance with JTG E30–2005 [26]. The specific steps are as follows: first, cure the 150 mm × 150 mm cylindrical specimens to 28 days of age and ensure the side surfaces are smooth and flat; use a rebound tester with a working length of 61.5 ± 0.3 mm for testing; evenly arrange 16 measuring points on the side surface of each specimen, avoiding the edges and any visible defects; eliminate the three maximum and three minimum rebound values to exclude abnormal data; calculate the average rebound value from the remaining 10 valid data points; convert the average rebound value to the rebound modulus using the calibration curve specified in the standard.

A three-factor, three-level orthogonal experimental design was adopted [28], with the water-to-binder ratio (0.35, 0.38, 0.42), recycled coarse aggregate replacement ratio (30%, 50%, 70%), and air-entraining agent dosage (0.02%, 0.04%, 0.06% by binder mass) as control variables. The experimental matrix was established accordingly, and multi-variable regression equations were developed to analyze the main and interaction effects of these factors on the mechanical and durability properties of RAC. Analysis of variance (ANOVA) was further performed to evaluate the statistical significance of each parameter, and the optimal mix combination satisfying both strength and durability requirements was determined based on the comprehensive evaluation results [29,30]. In addition, the core objective of the orthogonal experiment in this study is to screen out the optimal mix proportion of RAC that simultaneously meets the mechanical property, durability, and workability requirements for pavement bases. All evaluation indices (7-day compressive strength, permeability coefficient, and rebound modulus) are screened based on the corresponding specification limits.

#### 2.3.2. Freeze–Thaw Durability Test

To evaluate the freeze–thaw resistance of RAC under cold-region service conditions [31,32], specimens were subjected to rapid freeze–thaw cycling after 28 d of standard curing, following the procedure specified in GB/T 50082–2009 [27]. The water-freezing method was adopted, with temperature variations controlled between −18 °C and 5 °C for each cycle lasting 2–4 h. The test included 0, 100, 200, and 300 freeze–thaw cycles, and subsets of specimens were removed every 25 cycles for performance evaluation. Three main parameters were measured: (1) mass loss rate, reflecting surface scaling and internal damage; (2) relative dynamic modulus of elasticity, obtained using ultrasonic pulse velocity testing to assess internal structural degradation; and (3) compressive strength, determined through standard compression tests to evaluate load-bearing capacity after freeze–thaw exposure. All test results were compared with those of the uncycled reference specimens to characterize the degradation behavior and damage evolution of RAC under varying numbers of freeze–thaw cycles.

#### 2.3.3. Abbreviations

The abbreviation statistics table of this study is shown in Table 2.

## 3. Results and Discussion

### 3.1. Main Effect Analysis and Significance Test Based on Orthogonal Experiment

To efficiently and systematically evaluate the effects of three factors—A: water-to-binder ratio, B: recycled coarse aggregate replacement ratio, and C: air-entraining agent dosage—each at three levels, on the early mechanical and permeability properties of RAC, an orthogonal experimental design was adopted. This method enables the separation and quantitative assessment of the main effects of each factor within a limited number of tests, providing a reliable and efficient approach for practical mix design optimization. The Rc, K, and Ec were selected as the response indices. The orthogonal tests were conducted accordingly, and the results were analyzed through range analysis and to determine the relative significance of each factor and their influence trends [33,34]. The results are summarized in Table 3.

#### 3.1.1. Main Effects and Range Analysis

The results of the orthogonal design were further analyzed by calculating the mean response values for each factor level and subsequently determining the corresponding ranges. The mean values (ki) represent the average performance of a given factor at a specific level, while the range (R) quantifies the sensitivity of the response to the variation in that factor. A larger R value indicates a stronger influence on the target property. The results of the main effect and range analysis are summarized in Table 4.

As can be seen, for the Rc, the water–binder ratio exhibits the largest range (R = 4.70), indicating that it exerts the most pronounced effect, followed by the replacement ratio of recycled aggregate (R = 3.40), while the air-entraining agent dosage (R = 2.87) plays a relatively minor role. In terms of the K, the replacement ratio dominates with the highest range (R = 0.39), suggesting that increasing the proportion of recycled aggregate significantly increases the connectivity of pores and weakens the matrix resistance to water penetration. The effect of the water–binder ratio on permeability is also noticeable (R = 0.30), whereas the air-entraining agent has a smaller influence (R = 0.18), likely due to the balance between beneficial micro-air voids and the potential increase in porosity at excessive dosages. For the Ec, the replacement ratio again demonstrates the highest sensitivity (R = 1.93), highlighting the critical role of recycled aggregate stiffness and interface quality in governing the elastic response.

From a holistic perspective, the water–binder ratio is the most dominant factor for strength development, while the replacement ratio is decisive for both modulus and permeability. The air-entraining agent, although less influential on strength, provides secondary benefits by improving frost resistance through microstructural modification. Considering the simultaneous requirements of strength, durability, and constructability for base course applications, the optimal combination is determined as A_2_B_2_C_2_. This combination strikes a balance between maintaining sufficient compressive strength, controlling permeability within acceptable limits, and ensuring adequate stiffness, which is essential for long-term pavement performance.

#### 3.1.2. ANOVA and Determination of the Optimal Combination

To further verify the reliability of the main effect and range analyses, an ANOVA was conducted on the orthogonal test results [35]. The outcomes are summarized in Table 5. Statistical tests were performed for each response variable to evaluate the significance level of different factors and to clarify their relative influence on RAC performance.

As shown in Table 5, the water-to-binder ratio exhibited a highly significant effect on both the 7 d compressive strength and the rebound modulus, mainly due to its critical role in enhancing matrix compactness and promoting cement hydration. The recycled aggregate replacement ratio showed the most pronounced influence on the rebound modulus and permeability coefficient, as the adhered mortar and porous structure of recycled aggregates weakened the density and integrity of the ITZ. The air-entraining agent had a comparatively minor effect; however, it contributed positively to improving pore uniformity and enhancing freeze–thaw resistance, particularly reflected in the permeability and modulus results. Overall, the water-to-binder ratio was identified as the dominant factor governing strength development, while the replacement ratio primarily controlled stiffness and durability performance.

By integrating the results from Table 4 and Table 5, the optimal combination was determined to be A_2_B_2_C_2_, corresponding to a water-to-binder ratio of 0.38, recycled aggregate replacement ratio of 50%, and air-entraining agent dosage of 0.04%. Compared with other combinations, this mix not only ensures adequate load-bearing capacity for pavement base applications but also achieves superior durability and frost resistance, providing reliable technical support for the practical application of RAC in cold-region infrastructure.

### 3.2. Verification of the Optimal Mix Proportion

**Figure 1:**
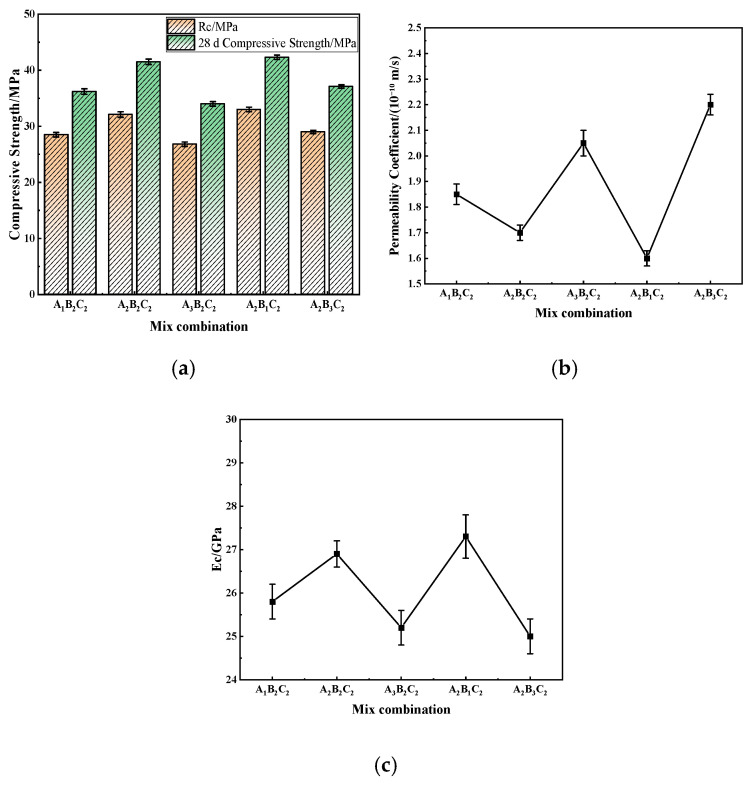
Verification of the optimal mix proportion: (**a**) Compressive strength; (**b**) Permeability coefficient; (**c**) Rebound modulus.

To verify the reliability of the optimal level combination A_2_B_2_C_2_ determined through the orthogonal test, comparative experiments were conducted with other representative mix combinations. The evaluation focused on three aspects—mechanical performance, permeability, and workability—to comprehensively assess the overall performance of the selected mix [36,37]. The corresponding results are presented in Figure 1.

As shown in Figure 1a, different mix proportions of recycled aggregate concrete (RAC) exhibited distinct strength variations at both 7 and 28 days. Overall, the compressive strength decreased with increasing water-to-binder ratio or recycled aggregate replacement ratio. The A_3_B_2_C_2_ mix showed the lowest performance, with 7-day and 28-day compressive strengths of only 26.8 MPa and 34.0 MPa, respectively. In contrast, the A_2_B_2_C_2_ mix (the optimal combination) achieved 32.1 MPa at 7 days and increased to 41.5 MPa at 28 days, significantly higher than the other groups. This indicates that a moderate water-to-binder ratio and replacement level can ensure sufficient hydration while mitigating the adverse effects of recycled aggregate defects, thereby achieving higher strength.

Figure 1b further illustrates the permeability results, which reveal differences in pore structure among the mixes. As the replacement ratio increased from 30% to 70%, the permeability coefficient rose from 1.60 × 10^−10^ m/s to 2.20 × 10^−10^ m/s, indicating that higher replacement levels markedly increased pore connectivity and interfacial defects. The A_2_B_2_C_2_ mix had a relatively low permeability coefficient of 1.70 × 10^−10^ m/s, suggesting a denser internal structure. The appropriate dosage of air-entraining agent in this mix played a beneficial role by forming uniformly distributed micro-air voids, which effectively offset the inherent porosity of recycled aggregates and reduced permeability.

Figure 1c presents the rebound modulus results for different mixes. The A_2_B_2_C_2_ mix achieved the highest modulus of 26.9 GPa, followed closely by A_2_B_1_C_2_ (27.3 GPa), indicating that a moderate replacement level helps maintain adequate stiffness and deformation compatibility. In contrast, mixes with higher water-to-binder ratios or replacement levels exhibited significantly lower rebound moduli, reaching as low as 25.0 GPa. This suggests that excessive water content increases matrix porosity and reduces stiffness, while excessive recycled aggregate content weakens the interfacial transition zone, leading to reduced elastic response.

In addition, the unexpected performance of mix A_1_B_2_C_2_—lower compressive strength, higher permeability and lower rebound modulus compared to mixes with higher water-to-binder ratios—stems from the synergistic effect of the low water-to-binder ratio and excessive air-entraining agent dosage. While air-entraining agents typically improve freeze–thaw resistance via micro-air voids, excessive dosage introduces excessive and partially connected bubbles that disrupt the cement paste compactness and weaken the aggregate-paste interfacial transition zone; compounded by the high viscosity and low fluidity of the low water-to-binder ratio paste, these bubbles are difficult to eliminate during mixing and compaction, leading to more internal defects.

Compared with the technical requirements for pavement bases specified in Technical Specifications for JTG/T F20–2015 [24] and JTG D40–2011 [38], the mix proportion A_2_B_2_C_2_ features a water-to-binder ratio of 0.38, a recycled aggregate replacement ratio of 50% and an air-entraining agent dosage of 0.04%. Its Rc is 32.1 MPa, 28 d compressive strength is 41.5 MPa, permeability coefficient is 1.7 × 10^−10^ m/s and rebound modulus is 26.9 GPa, all of which significantly exceed the specification limits of ≥30 MPa, ≥40 MPa, ≤2.0 × 10^−10^ m/s and ≥25 GPa, respectively. Additionally, the retention rates of all properties after 300 freeze–thaw cycles meet the required criteria—the relative dynamic modulus is no less than 70% and the mass loss rate is no more than 5%—fully adapting to the service demands of pavement bases. For other mix proportions: A_1_B_1_C_1_ has a water-to-binder ratio of 0.35, a replacement ratio of 30% and an air-entraining agent dosage of 0.02%. It meets the basic strength requirements but lacks sufficient comprehensive durability, as its relative dynamic modulus after freeze–thaw cycles is only 68%, below the specification limit of 70%. A_2_B_1_C_2_ has a replacement ratio of 30%, with slightly better mechanical properties but low utilization efficiency of recycled aggregates, which is inconsistent with the goal of circular economy. Therefore, A_2_B_2_C_2_ is determined as the optimal mix proportion.

### 3.3. Evolution of Mechanical Properties and Durability Under Freeze–Thaw Cycles

**Figure 2:**
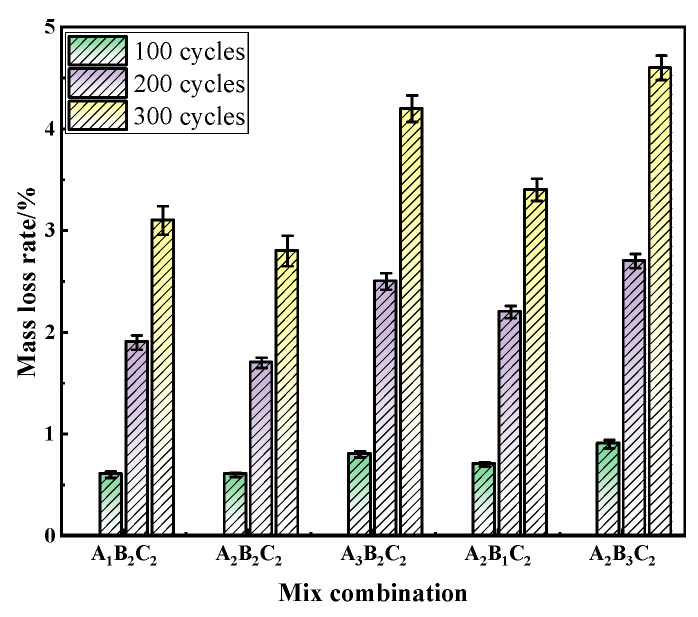
Mass loss rate of RAC under different freeze–thaw cycles.

**Figure 3:**
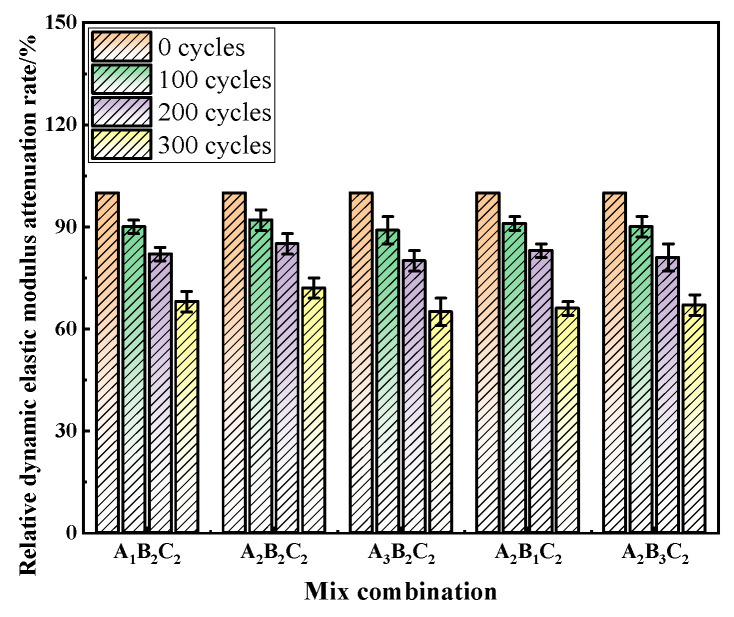
Relative dynamic elastic modulus attenuation rate of RAC under different freeze–thaw cycles.

**Figure 4:**
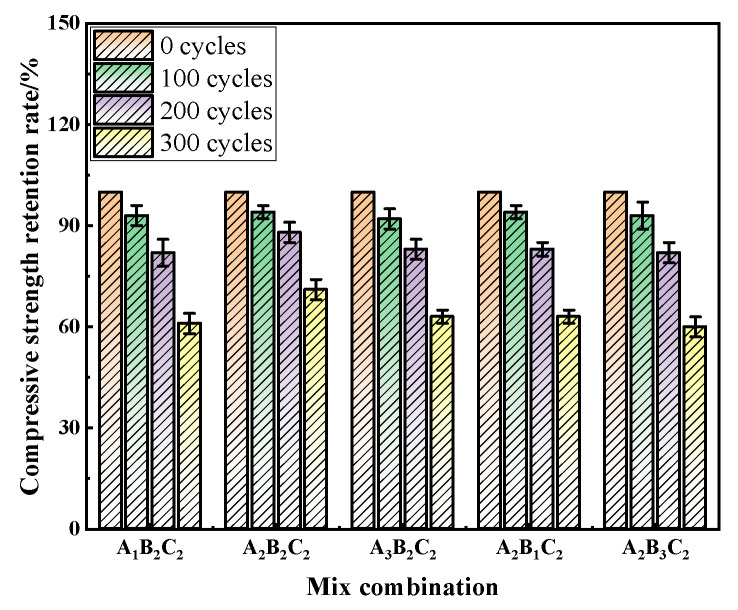
Compressive strength retention of RAC under different freeze–thaw cycles.

To evaluate the durability of RAC under cold-region service conditions, rapid freeze–thaw cycle tests were conducted within a temperature range of −18 °C to 5 °C, with cycle counts set at 0, 100, 200, and 300 [39,40]. At each stage, the specimens were tested for mass loss rate, relative dynamic elastic modulus, and compressive strength retention to characterize their degradation behavior. The corresponding results are presented in Figure 2, Figure 3 and Figure 4.

As shown in Figure 2, the mass loss rate of RAC increased progressively with the number of freeze–thaw cycles, although the magnitude of degradation varied significantly among different mix proportions. At the early stage (100 cycles), the mass loss of all groups remained below 0.5%, indicating that freeze–thaw damage was mainly limited to the surface mortar and a few weak aggregates, with negligible impact on the overall structure. After 200 cycles, the mass loss rate increased markedly, particularly for the A_2_B_3_C_2_ and A_3_B_2_C_2_ mixes, reaching 2.7% and 2.5%, respectively—much higher than that of the A_2_B_2_C_2_ mix (1.7%). This suggests that higher replacement ratios or water-to-binder ratios tend to form more connected capillary pores, increasing susceptibility to water ingress and frost-induced expansion. After 300 cycles, the A_2_B_3_C_2_ mix exhibited severe surface scaling with a mass loss of 4.6%, while the A_2_B_2_C_2_ mix showed only 2.8%, demonstrating a clear durability advantage. These results confirm that a well-balanced mix design can effectively mitigate surface deterioration caused by freeze–thaw action.

Figure 3 further illustrates the variation in relative dynamic modulus of elasticity. Taking the uncycled specimens as the reference, all mixes exhibited an exponential decay trend with increasing cycles. After 100 cycles, the modulus retention of all mixes remained above 90%, indicating limited internal damage. At 200 cycles, the A_2_B_2_C_2_ mix still retained 85% of its initial modulus, while the A_3_B_2_C_2_ and A_2_B_3_C_2_ mixes decreased to 80% and 81%, respectively. After 300 cycles, the optimal mix (A_2_B_2_C_2_) maintained 72% of its modulus, compared to only 65% for A_3_B_2_C_2_ and 68% for A_1_B_2_C_2_. These differences highlight the critical influence of mix design on the microstructural integrity of RAC. The moderate water-to-binder ratio in A_2_B_2_C_2_ produced a denser matrix with lower pore connectivity, while the appropriate air-entraining dosage generated uniformly distributed closed microbubbles that provided effective buffering space for internal frost expansion. Consequently, this mix exhibited significantly slower modulus degradation. In contrast, mixes with higher replacement ratios contained aggregates with higher porosity and adhered mortar, resulting in weaker interfacial zones and faster accumulation of freeze–thaw damage. It is worth noting that the air-entrained content of all mixtures was tested in accordance with GB/T 50080–2016 [41]. The results are as follows: A_1_B_1_C_1_ 3.2%, A_1_B_2_C_2_ 4.8%, A_2_B_2_C_2_ 3.5%, A_2_B_3_C_2_ 3.0%, A_3_B_2_C_2_ 2.8%. The optimal air-entrained content range for freeze–thaw resistance (3.0% to 4.0%) is achieved by mix A_2_B_2_C_2_, which explains its superior performance in resisting freeze–thaw damage—the uniformly distributed closed microbubbles effectively buffer internal frost expansion pressure without compromising matrix compactness.

The compressive strength degradation trend, as shown in Figure 4, reflects the mechanical stability of RAC under freeze–thaw exposure. After 100 cycles, all mixes retained 92–94% of their original strength, indicating minimal deterioration. At 200 cycles, differences among the mixes became more pronounced: the A_2_B_2_C_2_ mix maintained 88% of its initial strength, notably higher than the other mixes, which remained around 82–83%. After 300 cycles, the A_2_B_2_C_2_ mix still retained 71% of its strength—only a 15% reduction—whereas the A_2_B_3_C_2_ and A_3_B_2_C_2_ mixes dropped to 60% and 63%, respectively. These results demonstrate that the optimal combination maintained superior load-bearing capacity throughout freeze–thaw cycling and exhibited greater frost resistance than conventional concrete.

Considering all three parameters—mass loss rate, relative dynamic modulus, and compressive strength retention—the degradation process of RAC under freeze–thaw cycles can be divided into three distinct stages. The latent stage (0–100 cycles) is characterized by slow deterioration; the acceleration stage (100–200 cycles) involves increased pore connectivity and concentrated frost expansion, leading to rapid performance loss; and the stabilization stage (200–300 cycles) shows gradual saturation of internal damage and a slower rate of decline. Throughout this process, the A_2_B_2_C_2_ mix consistently demonstrated the best performance across all indices, confirming that the synergistic optimization of the water-to-binder ratio (0.38), replacement ratio (50%), and air-entraining agent dosage (0.04%) effectively compensated for the intrinsic defects of recycled aggregates and enhanced frost resistance. Overall, the optimized RAC not only satisfies the frost resistance requirements for engineering applications but also exhibits a slower degradation rate than conventional concrete, highlighting its strong potential for use in cold-region pavement base courses.

### 3.4. Comprehensive Performance Evaluation and Parametric Characterization of Degradation Mechanisms

Based on the freeze–thaw cycling tests described above, the relative dynamic modulus of elasticity and compressive strength retention of RAC under 0, 100, 200, and 300 cycles were obtained for different mix combinations. To further elucidate the underlying degradation mechanisms, the experimental data were analyzed through exponential regression and damage variable modeling [42,43], allowing a quantitative and parametric description of the deterioration process. The specific analytical procedure is outlined as follows:

First, the relative dynamic modulus (*Er*/*E*0) and compressive strength retention (*σc*/*σc*0) at different freeze–thaw cycles were normalized with respect to the uncycled reference specimens (*E*0, *σc*0). The natural logarithm of these normalized values was then taken to establish the linear regression models expressed as:

(1) $$\ln\left(\frac{E_{r}}{E_{0}}\right)=-kt+c,\;\;\;\ln\left(\frac{\sigma_{c}}{\sigma_{c0}}\right)=-k_{s}t+c^{’}$$

where t denotes the number of freeze–thaw cycles, c and c′ are regression constants reflecting the initial performance level of the material before freeze–thaw exposure. The degradation coefficients k and k_s_ are obtained by performing linear regression on the natural logarithm of relative dynamic modulus Er/E_0_ and relative compressive strength σc/σc0 against t using the least square method—specifically, k is the slope of the regression line for ln(Er/E_0_) versus t, and k_s_ is the slope of the regression line for ln(σc/σc0) versus t, both reflecting the rate of performance degradation under freeze–thaw cycles. The coefficient of determination R^2^ is a statistical indicator evaluating the goodness of fit of the regression model, calculated as R^2^ = 1 − Σ(yi − ŷi)^2^/Σ(yi − ȳ)^2^ where yi is the measured value of ln(Er/E_0_) or ln(σc/σc0), ŷi is the corresponding predicted value from the regression model, and ȳ is the average of the measured values.

Next, the damage variable (D(t)) was defined as follows:

(2) $$D\left(t\right)=1-\frac{E_{r}(t)}{E_{0}}$$

The values of *D*100, *D*200, *D*300 corresponding to 100, 200, and 300 freeze–thaw cycles, respectively, were calculated to quantitatively identify the “latent–acceleration–stabilization” stages of deterioration. Based on these models, the fitting results and calculated damage variables for each mix combination are summarized in Table 6 and Table 7.

As shown in Table 6 and Table 7, all mix combinations achieved coefficients of determination (R^2^) above 0.996, indicating that the exponential decay model accurately describes the freeze–thaw deterioration behavior of RAC. However, the degradation rates varied significantly among the mixes. The A_2_B_2_C_2_ combination exhibited the lowest decay coefficient (k = 1.097 × 10^−3^) and a damage variable of only 0.28 after 300 cycles, whereas A_3_B_2_C_2_ and A_2_B_3_C_2_ reached 0.35 and 0.33, respectively, reflecting a more accelerated degradation process. Similarly, the compressive strength decay coefficient (k_strength) was also lowest for A_2_B_2_C_2_ (1.254 × 10^−3^), with a strength retention of 71%, significantly higher than that of other mixes, which remained below 65%.

Mechanistically, these differences can be attributed to three synergistic effects: ① Dense matrix effect: A lower water-to-binder ratio reduces capillary pore connectivity, thereby slowing moisture migration; ② Interfacial balance effect: A 50% replacement ratio provides an optimal balance between mechanical integrity and aggregate defects, maintaining a relatively sound ITZ; ③ Air-entrainment buffering effect: An appropriate dosage of air-entraining agent generates uniformly distributed closed air voids, effectively relieving internal frost-induced pressure. The combined action of these effects enables A_2_B_2_C_2_ to exhibit the lowest degradation rate and minimal cumulative damage in the parametric characterization.

In summary, the normalization of retention data, logarithmic regression, and damage variable analysis together provide a quantitative framework for describing and interpreting the freeze–thaw deterioration of RAC. This parametric approach not only enhances the scientific rigor and interpretability of the experimental results but also offers a practical tool for predicting frost resistance and service life of RAC in engineering applications.

## 4. Conclusions

This study systematically investigated the performance requirements of road base layers in cold regions, focusing on the mix optimization, mechanical and permeability verification, and freeze–thaw durability evaluation of RAC. Combined with a parametric model, the deterioration patterns and mechanisms were revealed. The research not only verified the engineering applicability of the optimal mix but also provided theoretical support for the design and service life prediction of RAC.

(1) Through orthogonal tests and multi-index analysis, the water–binder ratio, replacement rate, and air-entraining agent were identified as key factors determining RAC performance. Among them, the water–binder ratio had the most significant influence on strength and permeability; the replacement rate mainly governed elastic modulus and interfacial stability; while the air-entraining agent markedly improved frost resistance. Considering both mechanical and durability requirements, the recommended mix is a water–binder ratio of 0.38, a replacement rate of 50%, and an air-entraining agent dosage of 0.04%.
(2) The optimal mix showed outstanding mechanical performance. The compressive strengths at 7 d and 28 d reached 32.1 MPa and 41.5 MPa, respectively; the rebound modulus was 26.9 GPa; and the permeability coefficient was only 1.7 × 10^−10^ m/s. These results were significantly superior to other combinations, confirming the rationality and applicability of the proposed mix design.
(3) Under freeze–thaw cycles, the RAC exhibited a typical three-stage degradation pattern of “latent period–acceleration period–stabilization period.” After 300 cycles, the dynamic modulus and compressive strength retention of the optimal mix remained at 72% and 71%, respectively, with a mass loss rate of only 2.8%. These values were markedly better than those of high replacement mixes and ordinary concrete, indicating excellent freeze–thaw resistance in cold regions.
(4) Exponential regression and damage variable analysis showed that the modulus attenuation coefficient k and strength attenuation coefficient k_strength of the optimal mix were the lowest, and the growth rate of the damage variable was the slowest. The superior performance was attributed to the dense matrix effect resulting from the low water–binder ratio, the preservation of interfacial integrity at a moderate replacement rate, and the buffering effect of uniformly distributed closed microbubbles formed by the air-entraining agent.

## Figures and Tables

**Table 1:** Basic properties of materials.

Material	Index	Unit	Value
P·O 42.5 Portland cement	Specific surface area	m^2^/kg	350
	Initial setting time	min	175
	28 d compressive strength	MPa	49.5
River sand (fine aggregate)	Fineness modulus	—	2.6
	Mud content	%	1.5
Natural crushed stone	Particle size range	mm	5–26.5
	Flaky particle content	%	8
	Crushing value	%	15
RCA	Bulk density	g/cm^3^	2.45
	Water absorption	%	5.8
	Flaky particle content	%	12
	Adhered mortar content	%	30
Lignosulfonate-based air-entraining agent	Solid content	%	5
Class II fly ash	Specific surface area	m^2^/kg	420
	Water demand ratio	%	95

**Table 2:** List of Abbreviations.

Abbreviation	Full Name
RAC	Recycled Aggregate Concrete
RCA	Recycled Coarse Aggregate
Rc	7d compressive strength
K	Permeability coefficient
Ec	Rebound modulus
w/b	Water-to-Binder Ratio
Er/E_0_	Relative Dynamic Modulus of Elasticity
σc/σc0	Relative Compressive Strength
R^2^	Coefficient of Determination

**Table 3:** Orthogonal experimental design and test results.

Number	A	B/%	C/%	Rc/MPa	K/10^−10^ m/s	Ec/GPa
1	0.35	30	0.02	36.2	1.45	28.1
2	0.35	50	0.04	34.8	1.62	27.5
3	0.35	70	0.06	32.5	1.88	26.9
4	0.38	30	0.04	34.7	1.55	27.3
5	0.38	50	0.06	33.1	1.72	26.8
6	0.38	70	0.02	31.6	1.95	26.2
7	0.42	30	0.06	31.2	1.78	26.5
8	0.42	50	0.02	29.8	1.96	25.8
9	0.42	70	0.04	28.4	2.12	25.3

**Table 4:** Main effect averages and range analysis.

Factor	Level	ki(Rc)/MPa	ki(K)/10^−10^ m/s	ki(Ec)/GPa
A	0.35	34.50	1.65	27.50
	0.38	33.13	1.74	26.77
	0.42	29.80	1.95	25.87
R (A)		4.70	0.30	1.63
B	30%	34.03	1.59	27.30
	50%	32.59	1.77	26.73
	70%	30.63	1.98	25.37
R (B)		3.40	0.39	1.93
C	0.02%	32.53	1.79	26.60
	0.04%	33.40	1.71	26.90
	0.06%	30.83	1.89	26.90
R (C)		2.87	0.18	0.30

**Table 5:** ANOVA analysis results.

Response	Source	SS	df	MS	F	*p* Value	Significance
Rc	A	40.21	2	20.105	55.87	<0.001	Highly significant
	B	12.45	2	6.225	17.31	0.002	Significant
	C	4.16	2	2.080	5.78	0.045	Slightly significant
	Error	2.14	2	1.070			
K	A	0.42	2	0.210	10.50	0.012	Significant
	B	0.37	2	0.185	9.25	0.015	Significant
	C	0.56	2	0.280	14.00	0.006	Significant
	Error	0.09	2	0.045			
Ec	A	7.25	2	3.625	42.18	<0.001	Highly significant
	B	6.34	2	3.170	36.87	<0.001	Highly significant
	C	2.16	2	1.080	12.57	0.008	Significant
	Error	0.87	2	0.435			

**Table 6:** Exponential regression results of relative dynamic elastic modulus.

Combination Type	k/×10^−3^	R^2^	D100	D200	D300
A_2_B_2_C_2_	1.097	0.998	0.080	0.150	0.280
A_2_B_1_C_2_	1.140	0.996	0.090	0.170	0.340
A_1_B_2_C_2_	1.227	0.997	0.100	0.180	0.320
A_2_B_3_C_2_	1.309	0.997	0.100	0.190	0.330
A_3_B_2_C_2_	1.358	0.997	0.110	0.200	0.350

**Table 7:** Exponential regression results of compressive strength.

Combination Type	k_Strength/×10^−3^	R^2^_Strength	Retention @300/%
A_2_B_2_C_2_	1.254	0.999	71
A_2_B_1_C_2_	1.339	0.997	63
A_1_B_2_C_2_	1.404	0.998	61
A_3_B_2_C_2_	1.432	0.997	63
A_2_B_3_C_2_	1.445	0.997	60

## Data Availability

The original contributions presented in this study are included in the article. Further inquiries can be directed to the corresponding authors.
